# A Genotoxic Stress-Responsive miRNA, miR-574-3p, Delays Cell Growth by Suppressing the Enhancer of Rudimentary Homolog Gene *in Vitro*

**DOI:** 10.3390/ijms15022971

**Published:** 2014-02-20

**Authors:** Ken-ichi Ishikawa, Atsuko Ishikawa, Yoshimi Shoji, Takashi Imai

**Affiliations:** Advanced Radiation Biology Research Program, Research Center for Charged Particle Therapy, National Institute for Radiological Sciences, Anagawa 4-9-1, Inage, Chiba 263-8555, Japan; E-Mails: k_ishi@nirs.go.jp (K.I.); a_ishi@nirs.go.jp (A.I.); y_otsuka@nirs.go.jp (Y.S.)

**Keywords:** irradiation, cell growth delay, miR-574-3p, enhancer rudimentary homolog gene

## Abstract

MicroRNA (miRNA) is a type of non-coding RNA that regulates the expression of its target genes by interacting with the complementary sequence of the target mRNA molecules. Recent evidence has shown that genotoxic stress induces miRNA expression, but the target genes involved and role in cellular responses remain unclear. We examined the role of miRNA in the cellular response to X-ray irradiation by studying the expression profiles of radio-responsive miRNAs and their target genes in cultured human cell lines. We found that expression of miR-574-3p was induced in the lung cancer cell line A549 by X-ray irradiation. Overexpression of miR-574-3p caused delayed growth in A549 cells. A predicted target site was detected in the 3′-untranslated region of the enhancer of the rudimentary homolog (*ERH*) gene, and transfected cells showed an interaction between the luciferase reporter containing the target sequences and miR-574-3p. Overexpression of miR-574-3p suppressed ERH protein production and delayed cell growth. This delay was confirmed by knockdown of ERH expression. Our study suggests that miR-574-3p may contribute to the regulation of the cell cycle in response to X-ray irradiation via suppression of ERH protein production.

## 1. Introduction

Genotoxic stress, including ionizing irradiation, triggers a variety of cellular responses such as growth delay, DNA repair, apoptosis, and senescence [1–3]. These responses are the result of activation, inactivation, interaction, or changes in the activity level of numerous proteins. Although many studies have indicated that the fate of irradiated cells is specific to the cell type, tumor type, irradiation type, and nature of the induced stress [1–4], the mechanism that regulates these cellular responses remains unclear.

Early cellular responses to genotoxic stress are characterized by transcriptional regulation of genes [5]. Previous reports suggest that the effect of irradiation on gene expression networks is mediated by induction and/or suppression of specific transcription factors [6–8]. In addition, during the last decade, small RNA molecules such as microRNAs (miRNAs) were demonstrated to play important roles in the regulation of gene expression in almost all vertebrates [9–12].

miRNAs are a family of non-coding RNA molecules consisting of approximately 22 nucleotides. miRNAs bind to complementary elements in the 3′-untranslated regions (UTRs) of target mRNAs to regulate gene expression, mainly at the post-transcriptional level via the cleavage or translational repression of their target mRNAs [13]. Recent studies have indicated that radiation causes alterations in the expression levels of many miRNA molecules in malignant cells of the brain [14], head and neck [14], lung [15], prostate [16], lymphocytes [17], and colon [18], in addition to normal fibroblasts [19]. Interestingly, of the known species of miRNAs, only a few have been reported as commonly expressed in multiple tissues [20], suggesting that radiation-induced changes in miRNA expression may be tissue- or cell-type specific.

To understand the mechanisms underlying the regulation of gene expression in cellular responses to a given stimulus, identification of the target mRNAs is important. Although many *in silico* studies have predicted miRNA targets, these data need to be verified by *in vitro* and *in vivo* experiments. To date, several studies have experimentally shown changes in target mRNA expression induced by radiation-responsive miRNAs in humans. For example, Girardi *et al.* found that Gamma ray irradiation induced the expression of miR-27a and subsequently suppressed the expression of its predicted target “ATM” in human lymphocytes [17], and they further demonstrated a direct interaction between the two [21]. Kwon *et al.* reported that radiation-inducible miR-193a-3p induced apoptosis in glioma cells by directly targeting Mcl-1, an anti-apoptotic Bcl-2 family member [22]. These findings highlight the importance of experimental studies aimed at determining the mechanisms underlying the regulation of gene expression by miRNA in response to genotoxic stress.

In this study, we found that X-ray irradiation of cells from human lung adenocarcinoma, brain medulloblastoma, and astrocytoma induced the expression of miR-574-3p, which, in turn, suppressed the production of the enhancer of rudimentary homolog (ERH) protein and delayed cell growth. These findings provide important insight into the cellular responses to X-ray irradiation.

## 2. Results and Discussion

### 2.1. Induction of miRNA Expression after Irradiation

**Figure 1.**
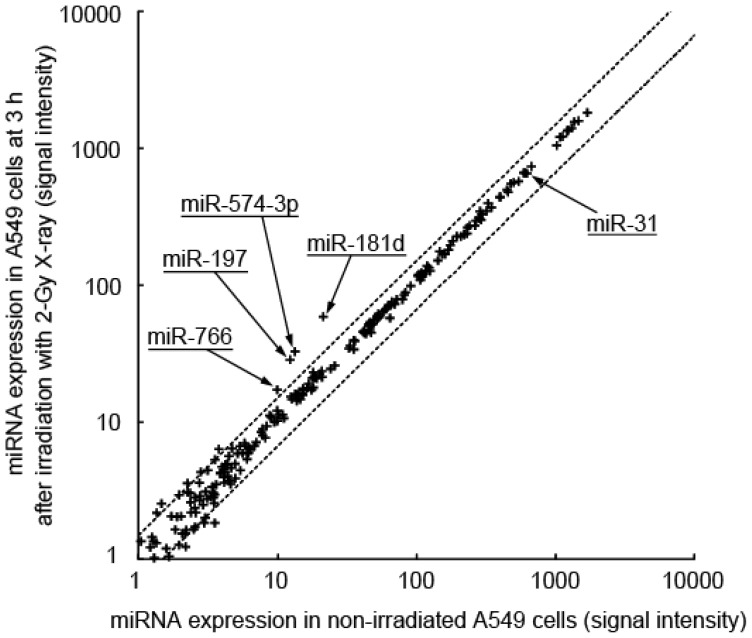
X-ray-responsive miRNA in A549 cells. The expression level of the miRNA in A549 cells after 3 h of irradiation with 2 Gy X-ray was compared with that of the miRNA in non-irradiated cells. The dotted lines indicate a threshold of 1.5-fold change.

**Figure 2.**
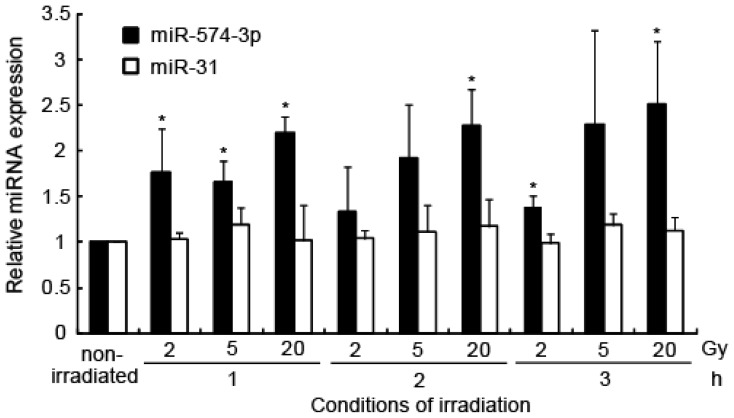
Induction of miR-574-3p after X-ray irradiation. Induction of miR-574-3p in A549 cells 1, 2, and 3 h after irradiation with 2, 5, and 20 Gy X-ray was determined by qRT-PCR, using the delta Ct method. miR-31 was used as a negative control. Error bars represent the standard deviation. Each experiment was conducted three times. * *p* < 0.05 *vs.* the non-irradiated sample.

To explore the role of miRNA molecules in X-ray-induced changes in gene expression, we analyzed the miRNA expression profile of the A549 cell line within a few hours of irradiation using miRNA arrays (Figure 1). After a 3-h exposure to 2-Gy X-irradiation, the cells showed induction of miR-181d (2.51-fold, *p* = 0), miR-574-3p (2.27-fold, *p* = 2.4 × 10^−34^), miR-197 (2.12-fold, *p* = 2.9 × 10^−26^), and miR-766 (1.64-fold, *p* = 2.5 × 10^−7^). Among the four miRNAs, induction of miR-574-3p (1.67-fold, *p* = 0.0011) and miR-766 (1.54-fold, *p* = 0.0001) was confirmed by quantitative RT-PCR (qRT-PCR). We did not find any downregulation of miRNA following exposure to 2-Gy irradiation. The irradiation-induced increase in miR-574-3p expression was further confirmed by qRT-PCR after 1, 2, and 3 h of exposure to 2, 5, and 20 Gy of X-irradiation (Figure 2). Because an interaction between miR-766 and its predictive target mRNA was not experimentally detected (data not shown), we focused on the characterization of miR-574-3p.

miR-574-3p was first identified in colorectal cancer cell lines [23]. miR-574-3p is downregulated in patients with bladder cancer [24], whereas it is upregulated in patients with hepatocellular carcinoma [25]. Because this miRNA displays differential expression regulation, we analyzed the response of miR-574-3p to X-irradiation in ONS-76 (brain medulloblastoma), SF126 (brain astrocytoma), C32TG (amelanotic melanoma), HeLa (cervical adenocarcinoma), and NB1RGB (normal skin fibroblast) cell lines. qRT-PCR analysis revealed that the irradiation increased miR-574-3p expression in A549 (2.5-fold, *p* = 0.0190), ONS76 (1.3-fold, *p* = 0.0469), and SF126 (1.2-fold, *p* = 0.0115) cell lines, whereas expression was unaffected in C32TG (1.0-fold, *P* = 0.4331) cells. Expression of miR-574-3p was significantly, although slightly, suppressed in the NB1RGB (0.9-fold, *p* = 0.0048) and HeLa (0.9 fold, *p* = 0.0377) cell lines.

Next, we attempted to find common genetic features in cell lines showing induction of miR-574-3p expression. *P53* mutations have been reported only in the ONS76 and SF126 cell lines [5], and the retinoblastoma 1 (*RB1*) mutation was shown only in the SF126 cell line [26,27]. Therefore, neither the *P53* nor the *RB1* loci present a common feature in the three cell lines that showed irradiation-induced up-regulation of miR-574-3p. Interestingly, the three cell lines all have a common homozygous deletion of the *CDKN2A* locus [26,27]. In contrast, the wild-type *CDKN2A* locus was reported to be present in the C32TG and HeLa cell lines, which do not show induction of miR-574-3p expression following irradiation [26–28]. The remaining cell line, NB1RGB, was established from normal human fibroblasts, and no information is available regarding the *CDKN2A* locus in public databases such as COSMIC [26,27] or IGRhCellID [28].

### 2.2. Delay in Cell Growth by Overexpression of miR-574-3p

**Figure 3.**
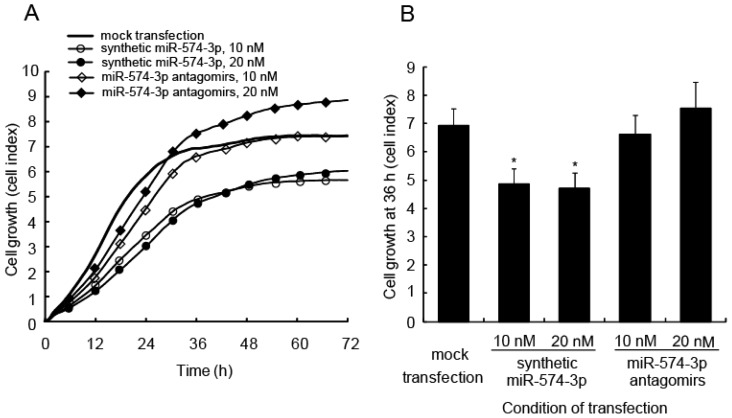
Delay in cell growth caused by overexpression of miR-574-3p in A549 cells. (**A**) A549 cells were transfected with 10 or 20 nM synthetic miR-574-3p or miR-574-3p antagomirs. Cells transfected with the synthetic miR-574-3p or miR-574-3p antagomirs were incubated for 24 h and then re-plated into the wells of a device that allows real-time measurement of cell growth (calculated as the cell index) at 1-h intervals. Each experiment was conducted four times; (**B**) Cell growth was assessed using the cell index at 36 h. * *p* < 0.05 *vs*. mock-transfected cells.

To study the biological significance of miR-574-3p induction, synthetic miR-574-3p or locked nucleic acid (LNA)-modified oligonucleotides (antagomirs) targeting miR-574-3p were transfected into A549 cells. Real-time monitoring of cell viability using an RT-CES instrument showed a significant delay in growth in the miR-574-3p-overexpressing cells (Figure 3A). Cell growth at 36 h was analyzed statistically (Figure 3B). Cells transfected with the synthetic miR-574-3p had a significant delay in growth compared with the cells transfected with antagomirs or compared with mock-transfected cells. Interestingly, cells transfected with antagomirs at 20 nM tended to show increased growth at later time points compared with the mock-transfected cells.

**Figure 4.**
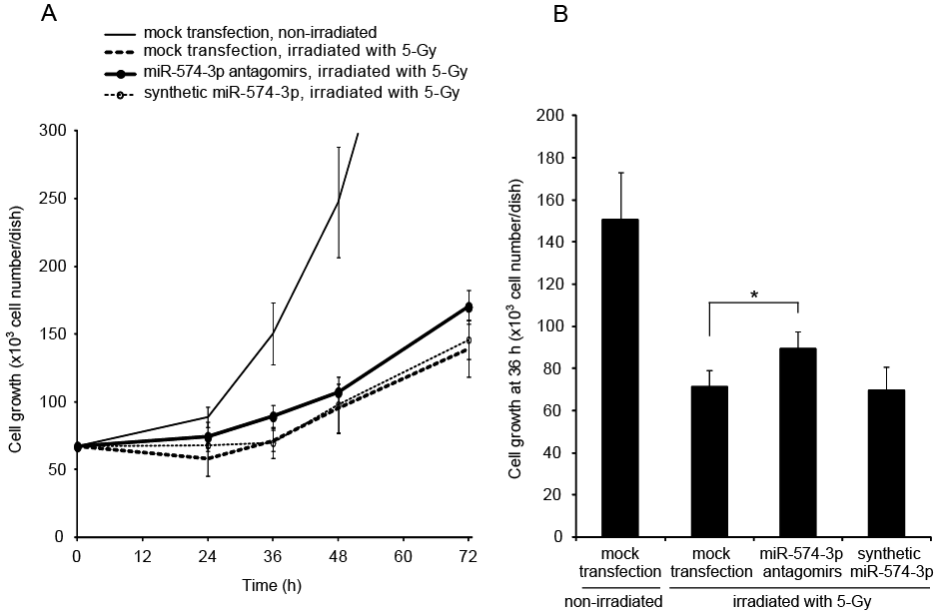
Attenuation of X-ray-induced growth delay in A549 cells through suppression of miR-574-3p. (**A**) A549 cells were transfected with 20 nM miR-574-3p antagomirs or synthetic miR-574-3p. Cells transfected with the miR-574-3p antagomirs or synthetic miR-574-3p were incubated for 24 h and then irradiated with 5-Gy of X-ray. X-irradiated cells were seeded on 35-mm dishes at a density of 67,000 cells per dish. Cells were counted using a hemocytometer at 24, 36, 48, or 72 h after irradiation. Each experiment was conducted three times; (**B**) Cell growth was assessed using the cell number at 36 h. * Statistical difference with *p* < 0.05 was considered significant.

Growth delay is a known cellular response to irradiation. To study the involvement of miR-574-3p in the growth delay induced by X-irradiation, the growth of A549 cells transfected with miR-574-3p antagomirs or synthetic miR-574-3p and subsequently irradiated with X-ray was observed. The growth delay induced by X-irradiation was partially attenuated in cells transfected with miR-574-3p antagomirs (Figure 4A). In contrast, the growth delay induced by X-irradiation did not increase in cells transfected with synthetic miR-574-3p. Therefore, the expression of miR-574-3p induced by 5-Gy of X-ray appears to be sufficient to delay growth in irradiated cells. At 36 h after X-irradiation, the number of cells in the miR-574-3p antagomirs group was higher than that in the mock-transfected group (1.3-fold, *p* = 0.0015) (Figure 4B).

### 2.3. Expression Analysis of Transcripts Regulated by miR-574-3p

**Figure 5.**
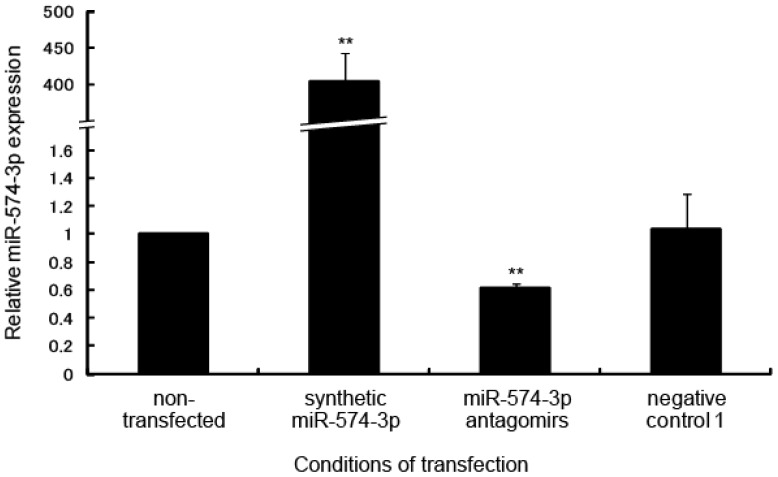
Overexpression or downregulation of miR-574-3p in A549 cells transfected with synthetic miR-574-3p or miR-574-3p antagomirs. Expression levels of miR-574-3p in A549 cells transfected with synthetic miR-574-3p or miR-574-3p antagomirs were determined by qRT-PCR. ** *p* < 0.01 *vs*. non-transfected cells.

To identify the target mRNA regulated by miR-574-3p, the expression profile of mRNA in A549 cells transfected with synthetic miR-574-3p was compared with that in cells transfected with miR-574-3p antagomirs. After transfection (24 h), the miR-574-3p expression level in each cell line was measured by qRT-PCR. As expected, compared to non-transfected cells, cells transfected with synthetic miR-574-3p showed significantly higher levels of miR-574-3p (404-fold, *p* = 5.2 × 10^−55^), whereas those transfected with the antagomirs showed considerably lower levels of miR-574-3p (0.61-fold, *p* = 0.0016) (Figure 5).

Using these transfection conditions, we utilized an mRNA array to identify 53 mRNAs that were downregulated by at least 0.5 fold (*p* ≤ 0.001) in cells overexpressing miR-574-3p compared to cells with miR-574-3p knocked down. Although various algorithms exist for miRNA target prediction [29–33], the predicted targets generated by these different prediction tools overlap poorly with the small number of validated targets [34]. Algorithms for miRNA targeting of mRNA have not been fully examined yet and are based mainly on experimentally validated miRNA-mRNA interactions, which represent only a portion of the possible interactions available *in vivo*. Therefore, experimental validation of predicted target sites is essential for studies of miRNA function. In this study, miRanda, a target-prediction tool, was selected because it is superior in predicting sites with imperfect binding between the miRNA and mRNA, although the rate of false positives is high [34].

Among the 53 targets identified, 11 genes had the predicted target sequences for miR-574-3p in their 3′-UTR. We assessed these target sequences using a BLAST search against the human genome. Three candidate genes were excluded from analysis because their target sequences contain repetitive sequences such as di-nucleotide repeats; however, this does not mean these repetitive sequences are not functional. Each of the remaining eight genes harbored a unique sequence in the 3′-UTR that is possibly targeted by miR-574-3p (Table 1). Gene ontology analysis revealed that among these eight genes, only *ERH* is associated with cellular responses to genotoxic stress.

The enhancer of rudimentary (*ER*) gene was originally isolated from *Drosophila melanogaster* and found to participate in the pyrimidine metabolic pathway [35]. Its human homolog, *ERH*, was identified later, and the nucleotide identity between the human and *Drosophila* genes is almost 80% [36]. Furthermore, the *ER* gene is highly conserved across animals, plants, and protists, but not fungi [37,38]. ERH is a multifunctional nuclear protein that mediates cell cycle progression [37,39] and transcriptional regulation [35,40]. Recently, Fujimura *et al.* reported that ERH-depleted cells showed severe chromosome misalignment and weakened kinetochore-microtubule attachment, followed by dissociation of the centromere-associated protein E (CENP-E), a mitotic kinesin that is involved in stabilizing the kinetochore-microtubule attachment [41]. Weng *et al.* also reported that ERH is required for mRNA splicing of CENP-E, in addition to localization of CENP-E at the kinetochore [42].

**Figure 6.**
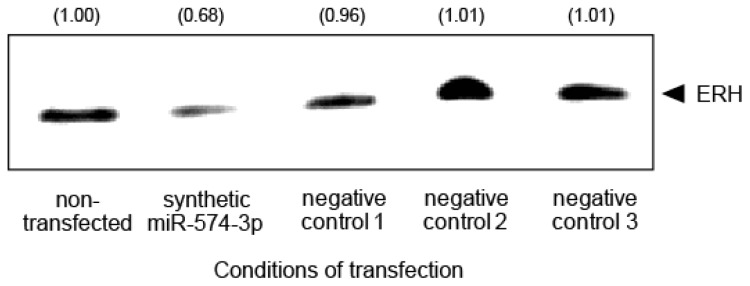
Downregulation of ERH in A549 cells overexpressing miR-574-3p. ERH protein in A549 cells transfected with synthetic miR-574-3p was detected by western blot analysis. The relative expression levels of the ERH protein in the cells are quantified and indicated in the panel.

*ERH* showed the highest hybridization intensity among the eight genes in our study, leading us to focus further on the *ERH* gene. To test whether ERH expression is affected by miR-574-3p, the miRNA was transfected into A549 cells. Western blot analysis revealed that expression of the ERH protein was 0.68 fold lower in A549 cells transfected with the synthetic miR-574-3p than in the non-transfected cells (Figure 6).

### 2.4. Binding of miR-574-3p to the 3′-UTR of ERH

**Figure 7.**
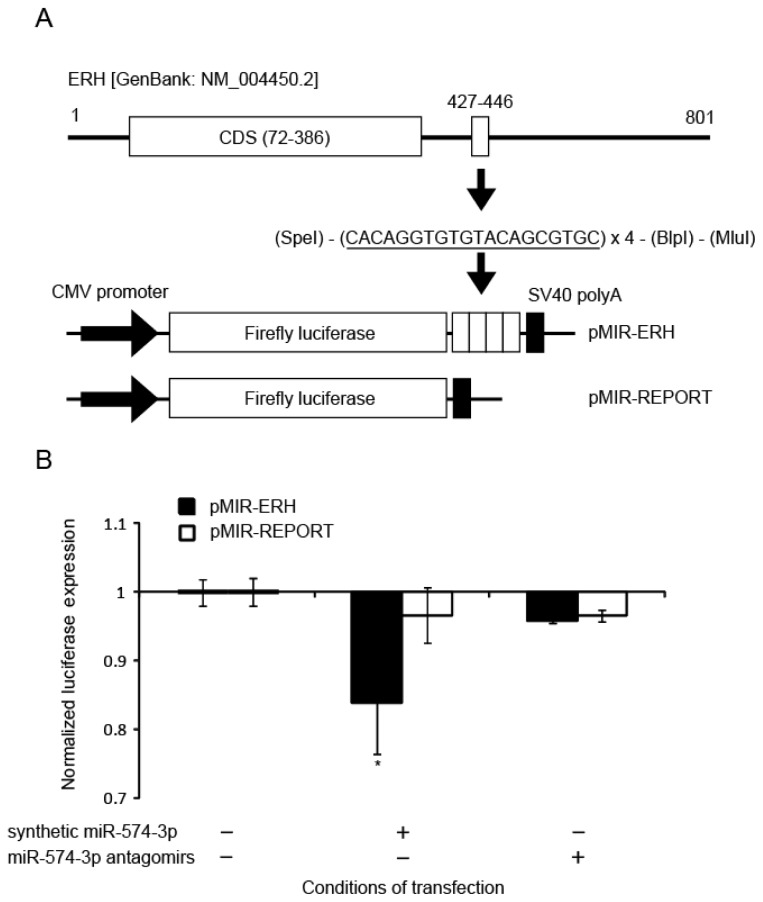
Luciferase reporter assay for binding of the modified ERH-3′-UTR with miR-574-3p. (**A**) The miR-574-3p target sequence was predicted to be at nucleotide positions 427 to 446 (3′-UTR) in *ERH* [Genbank: NM_004450.2]. The putative miR-574-3p recognition sequence (solid lines) was cloned with four repetitions downstream of the firefly luciferase gene in the pMIR-REPORT vector. Expression of the luciferase gene is controlled by the CMV promoter and SV40 polyA; (**B**) A549 cells were transfected with the pMIR-ERH plasmid or pMIR-REPORT vector without the ERH sequence as a control at 25 pM and 6 h later with synthetic miR-574-3p or the miR-574-3p antagomirs at 10 nM. Luciferase activity was measured 6 h after transfection with the synthetic miR-574-3p or miR-574-3p antagomirs. Each experiment was conducted three times. Error bars represent the standard deviation. * *p* < 0.05 *vs*. cells transfected with the pMIR-ERH vector.

To examine whether miR-574-3p could bind to the predicted sequence found in the 3′-UTR of the *ERH* mRNA, an 80-bp DNA fragment consisting of the four units of the predicted binding sequence (20 bp) of miR-574-3p was synthesized and cloned into the pMIR-REPORT Luciferase vector (Figure 7A). This construct, named pMIR-ERH, or the pMIR-REPORT vector without the ERH sequence as a control, was co-transfected into A549 cells with either synthetic miR-574-3p or miR-574-3p antagomirs. We found that the luciferase activity in A549 cells co-transfected with the synthetic miR-574-3p and the pMIR-ERH vector was significantly lower (0.84-fold, *p* = 0.0204) than that in the cells transfected with the pMIR-ERH vector, demonstrating that miR-574-3p bound to the target sequences (Figure 7B). These data suggest that miR-574-3p could regulate ERH function.

### 2.5. ERH Regulates Cell Cycle Progression

**Figure 8.**
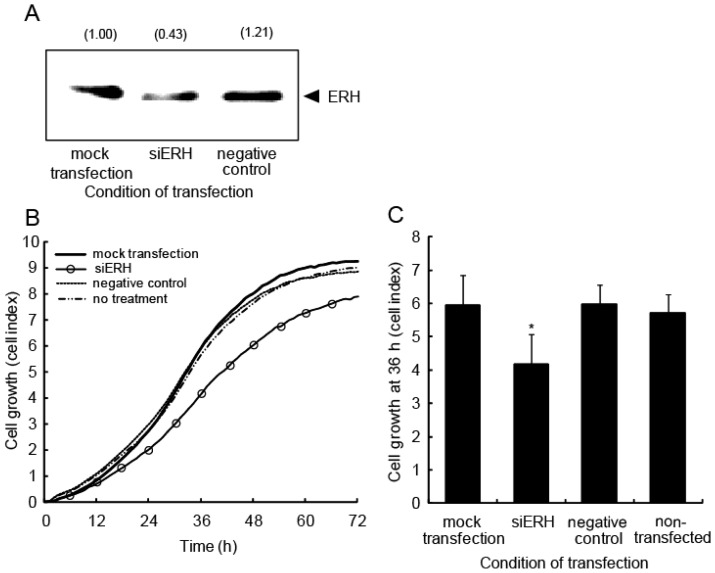
Cell growth is delayed when ERH is knocked down in A549 cells. (**A**) A549 cells were transfected with siRNA oligonucleotides for either ERH or a negative control at 5 nM. Total cell extracts were prepared 48 h after transfection. The expression level of ERH was confirmed by western blot analysis. The relative expression level of ERH protein was quantified and is indicated in the panel; (**B**) A549 cells transfected with siRNA for ERH were incubated for 24 h and then re-plated into the wells of a device for RT-CES. Each experiment was conducted four times; (**C**) Cell growth was assessed using the cell index at 36 h. * *p* < 0.05 *vs*. mock-transfected cells.

To ascertain whether ERH directly affects cell growth, siRNA against *ERH* (siERH) or negative control oligonucleotides were transfected into A549 cells. Western blot analysis showed that ERH expression was, as expected, lower in cells transfected with the siRNA (0.43-fold) than in those transfected with the negative control oligonucleotides and mock-transfected cells (Figure 8A). Real-time monitoring of cell viability using an RT-CES instrument revealed a significant growth delay in ERH-suppressed cells (Figure 8B). The cell growth at 36 h was analyzed statistically (Figure 8C). Cells transfected with siERH displayed a significant delay in growth compared with cells transfected with the negative control oligonucleotides, mock transfected cells, or non-transfected cells.

**Figure 9.**
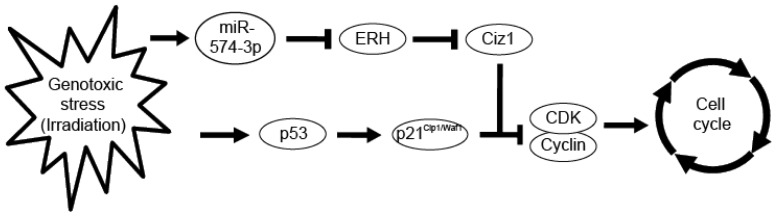
Proposed role of miR-574-3p in radiation-induced growth delay. Genotoxic stress, including irradiation, arrests the cell cycle through activation of p53 and subsequent induction of p21^Cip1/Waf1^, which binds to and inhibits CDK-cyclin complexes [43]. miR-574-3p is also induced by irradiation and suppresses the expression of ERH, which is the binding partner of Ciz1 [36,50]. Ciz1 is thought to support the CDK-cyclinE-p21^Cip1/Waf1^ complex. ERH blocks the action of Ciz1, but miR-574-3p induced by DNA damage reduces the expression of ERH, thus facilitating the formation of the CDK-cyclinE-p21^Cip1/Waf1^ complex. As a result, growth delay is maintained, thereby enabling DNA damage repair.

DNA damage caused by genotoxic stress is known to arrest cell cycle progression through the activation of the tumor suppressor protein, p53, and subsequent induction of the cyclin kinase inhibitor, p21^Cip1/Waf1^, which binds to and inhibits CDK-cyclin complexes [43]. In addition to these important components involved in cell cycle control, many proteins also interact with p53 and p21^Cip1/Waf1^. Mitsui *et al.* isolated the Cip1-interacting zinc finger protein, Ciz1, which binds to the *N* terminus of p21^Cip1/Waf1^ [44]. Ciz1 is aberrantly expressed in various types of tumors [45–48] and is thought to be a tumor suppressor [49]. Ciz1 is believed to facilitate the formation of the CDK-cyclinE-p21^Cip1/Waf1^ complex. Ciz1 induces the cytoplasmic localization of p21^Cip1/Waf1^ when it is upregulated by DNA damage. Therefore, we infer that the CDK-cyclinE-p21^Cip1/Waf1^ complex is formed under the action of Ciz1 to arrest the cell cycle after DNA damage. Interestingly, Ciz1 has been characterized as a novel molecular partner for human ERH [36,50]. We postulate that because ERH blocks the action of Ciz1, induction of miR-574-3p by DNA damage with subsequent reduction of ERH expression facilitates the formation of the CDK-cyclinE-p21^Cip1/Waf1^ complex. As a result, growth delay is maintained, thereby enabling the repair of DNA damage (Figure 9).

### 2.6. miR-574-3p Expression during Cellular Response to X-Ray Irradiation

In this study, miR-574-3p expression was induced by X-ray irradiation in cells harboring a homozygous deletion of the *CDKN2A* locus. The *CDKN2A* gene is among the most commonly mutated loci in human cancers [51] and encodes two different tumor suppressors, *P16^INK4a^* and *P14^ARF^*, which are translated from alternatively spliced mRNAs. *P16^INK4a^* is involved in cell cycle arrest and the induction of senescence [52], and it inhibits *CDK4/6*, thus keeping RB1 in its hypo-phosphorylated active state [52]. Hypo-phosphorylated RB1 suppresses G1 advance by binding to E2F transcription factors (inactive E2Fs). After RB1 is phosphorylated, the repression of E2F is released, driving the expression of genes required for the G1/S-phase transition and initiation of DNA synthesis [53,54]. In general, the loss of *P16^INK4a^* has been suggested to facilitate aberrant cell cycle progression through the RB1 pathway [55]. *P14^ARF^* is involved in the p53 pathway. By inhibiting MDM2, P14^ARF^ stabilizes and activates P53 [52], subsequently halting the cell cycle. *P14^ARF^* also functions independently of *P53*. In *P53*-deficient human tumor cell lines, expression of *P14^ARF^* arrests the cells in S or G2 phase, followed by an apoptotic response [56–60]. As *CDKN2A* encodes two proteins, the interpretation of the effect of the loss of the *CDKN2A/P16^INK4a^/P14^ARF^* locus is not straightforward. In this study, miR-574-3p expression was not induced in X-ray-irradiated cells harboring a normal *CDKN2A* locus. Rather, *P16^INK4a^* and *P14^ARF^* probably function in cells harboring a normal *CDKN2A* locus after X-ray irradiation. Although homozygous deletion of the *CDKN2A/P16^INK4a^/P14^ARF^* locus simultaneously compromises the function of both *P53* and *RB1*, we have not yet analyzed the association between deletion of the *CDKN2A/P16^INK4a^/P14^ARF^* locus and induction of miR-574-3p by X-ray irradiation. Although further studies are necessary to elucidate the precise mechanism of miR-574-3p and its target gene ERH, our finding lends important insight to the cellular response to X-ray irradiation.

## 3. Experimental Section

### 3.1. Cell Lines, Culture Conditions, and Irradiation

The following human cell lines were used in this study: A549 (lung adenocarcinoma), C32TG (amelanotic melanoma), ONS-76 (brain medulloblastoma), SF126 (brain astrocytoma), HeLa (cervical adenocarcinoma), and NB1RGB (normal skin fibroblasts). The A549 and C32TG cell lines were obtained from the Japanese Cancer Research Resource Bank (Osaka, Japan). The ONS-76 and SF126 cell lines were obtained from the Institute for Fermentation (Osaka, Japan). The HeLa and NB1RGB cell lines were obtained from the RIKEN Cell Bank (Ibaraki, Japan). The properties of the cell lines, including p53 mutations, have been described previously [5]. All cell lines were cultured in Eagle minimum essential medium (E-MEM; Nissui, Tokyo, Japan) supplemented with 10% fetal bovine serum (FBS; Biological Industries Ltd., Beit HaEmek, Israel) and grown in 5% CO_2_ at 37 °C. Cells were irradiated with 2, 5, or 20 Gy X-rays, as described previously, at a rate of approximately 1 Gy/min [61].

### 3.2. Isolation of Total RNA

Total RNA, including small RNA species, was isolated from the cells by using a mirVana miRNA isolation kit according to the manufacturer’s instructions (Life Technologies, Carlsbad, CA, USA). Total RNA was then quantified using RiboGreen Reagent (Life Technologies).

### 3.3. Transfection

The synthetic miRNA “miCENTURY OX miNatural” and antagomirs “miRCURY LNA Knockdown” were obtained from Exiqon (Vedbaek, Denmark). A549 cells were transfected with the oligonucleotides at 3, 10, or 20 nM using LipoTrust EX Oligo reagent, according to the manufacturer’s protocol (Hokkaido System Science, Hokkaido, Japan). Total RNA was extracted from the cells 24 h after transfection using a mirVana miRNA isolation kit. For western blot analysis, the cell extracts were collected from the cells at 60 h post-transfection by adding 2% SDS (sodium dodecyl sulfate), 10% glycerol, and 50 mM Tris-HCl (pH 6.8) supplemented with 100 mM DTT. The concentration of total protein in the cell extracts was measured using the bicinchoninic acid (BCA) method using the BCA Protein Assay Kit (Thermo Fisher Scientific, Rockford, IL, USA).

siRNA against ERH was obtained from Life Technologies. As a negative control for siRNA transfection, we used commercially designed Silencer Select Negative Control #2 (Life Technologies). A549 cells were transfected with the siRNA or Silencer Select Negative Control #2 at 5 nM using LipoTrust EX Oligo reagent. Cell extracts were collected 48 h post-transfection, and protein concentrations were measured as described earlier.

### 3.4. Microarray Analysis of miRNA Expression

miRNA expression analysis was conducted using Human miRNA Oligo Microarray, G4470A, and an miRNA Labeling Reagent & Hybridization Kit, according to the manufacturer’s instructions (Agilent Technologies, Santa Clara, CA, USA) [62]. This array consists of 470 human miRNA genes listed in the miRBase, release 9.1 [63–65], and contains 20–40 features targeting each of the 470 miRNA, although there are 4449 human miRNA genes listed in the latest version of miRBase, release 20. Once hybridized, the array was scanned using an Agilent DNA Microarray Scanner (Agilent Technologies), and TIFF images were fed into Feature Extraction software version 9.5.3 (Agilent Technologies) to extract the expression profile data. The data were further processed for statistical analysis using the advanced analysis tool Rosetta Resolver Gene Expression Data Analysis System, version 7.1 (Rosetta Biosoftware, Seattle, WA, USA). The Rosetta Resolver System calculates the expression data, *i.e.*, intensity and ratio, using an error-weighting algorithm. The error-weighting involves using measurement error derived from the microarray technology-specific error model and weighting expression measurements based on that error such that intensities from array probes with larger errors are given lower weight than intensities from array probes with smaller errors. Expression data were generally accepted as true when the *p*-value of each intensity and the ratio of the intensity in the overexpressed sample to that in the suppressed sample (log10 transformed) were ≤0.001. miRNA with ratios (overexpressed sample/suppressed sample) of at least 1.50 fold were considered hits, *i.e.*, induced miRNA. The gene expression omnibus accession number of the microarray data is GSE30130.

### 3.5. Quantitative Real-Time Reverse Transcription (RT)-PCR Analysis of miRNA Expression

For the reverse-transcription reaction, 1 μg of total RNA was used in conjunction with a miScript Reverse Transcription Kit (Qiagen Inc., Valencia, CA, USA). Quantitative real-time PCR was performed using the miScript System (Qiagen Inc.), comprising a miScript SYBR Green PCR Kit and miScript Primer Assay. A LightCycler 480 Real-Time PCR System (Roche Diagnostics, Basel, Switzerland) with a 384-well plate format was used. Each sample was run in triplicate.

### 3.6. Microarray Analysis of mRNA Expression

For mRNA expression analysis, a dual-color microarray-based gene expression analysis was conducted using the Whole Human Genome Oligo DNA microarray, G4112F, according to the manufacturer’s instructions (Agilent Technologies). Briefly, 1 μg of the total RNA sample from A549 cells, transfected with either the precursor oligonucleotides or the knockdown oligonucleotides, was amplified and labeled with either a Cy5 or Cy3 dye. This microarray consists of 41,000 unique probes. Once hybridized, the microarray was scanned, the expression profile was extracted, and the data were statistically analyzed using the Rosetta Resolver System. Expression data were accepted as true when the *p*-value of the overexpressed/suppressed ratio (log10 transformed) was ≤0.001. For this analysis, ratio values of <0.5 fold with intensities of the knocked down sample ≥20 were accepted as hits, *i.e.*, suppressed mRNA. The gene expression omnibus accession number of the microarray data is GSE30131.

### 3.7. Quantitative Real-Time RT-PCR Analysis of ERH Expression

To confirm the expression levels of the *ERH* mRNA, 1 μg of total RNA was reverse-transcribed using a Transcriptor First-Strand cDNA Synthesis Kit, according to the manufacturer’s instruction (Roche Diagnostics). *ERH* expression was quantified using a Light Cycler 480 Probe Master and Universal Probe Library (Roche Diagnostics). The LightCycler 480 Real-Time PCR System was also used. Each sample was analyzed three times.

### 3.8. Prediction of miR-574-3p Targets in Silico

The mature miRNA sequence of miR-574-3p was obtained from miRBase [66]. The 3′-UTR sequences of the candidate mRNAs were obtained from NCBI [67]. The putative miR-574-3p recognition elements of the candidate genes were predicted using the miRanda algorithm [29,30,68]. The identified putative elements were further subjected to a BLAST search [69] against the human genome to remove elements that match completely with more than two loci in the genome.

### 3.9. Western Blotting

Western blotting analysis was performed as described previously [70]. An ERH monoclonal antibody (MO7), clone 1H4 (Abnova, Taipei City, Taiwan), was used to detect ERH protein. The ERH protein was detected as a band of approximately 12 kDa.

### 3.10. Reporter Constructs

To generate a reporter vector bearing the miRNA-binding sites, the putative miR-574-3p recognition sequence in the 3’-UTR region of the *ERH* mRNA was synthesized with four repetitions and inserted downstream of the firefly luciferase gene in the pMIR-REPORT Luciferase vector (Life Technologies), as described previously [71]. Briefly, the oligonucleotides sequences were designed to carry the MluI and SpeI sites in the multicloning site of the pMIR-REPORT luciferase vector. The oligonucleotides used in these studies were as follows: 5′-CTAGTCACAGGTGTGTACAGC GTGCCACAGGTGTGTACAGCGTGCCACAGGTGTGTACAGCGTGCCACAGGTGTGTACAGCGTGCGCTGAGCA-3′ and 5′-CGCGTGCTCAGCGCACGCTGTACACACCTGTGGCACGCTGTA CACACCTGTGGCACGCTGTACACACCTGTGGCACGCTGTACACACCTGTGA-3′. A BlpI site (underlined) was added to each insert to test for positive clones.

### 3.11. Luciferase Assay

To investigate the effect of miR-574-3p on the expression of the ERH protein, cells were co-transfected with the pMIR-ERH construct at 25 pM and the pGL4.75[hRluc/CMV] construct at 2.5 pM (Promega Corporation, Madison, WI, USA) using the FuGENE HD transfection reagent according to the manufacturer’s protocol (Roche Diagnostics). At 6 h after transfection of the constructs, the oligonucleotides, namely, the synthetic miR-574-3p or miR-574-3p antagomirs, were transfected at 10 nM using the LipoTrust EX Oligo reagent, according to the manufacturer’s protocol (Hokkaido System Science). Six hours after oligonucleotide transfection, the cells were lysed using a 1× passive lysis buffer, and the activity of both firefly and Renilla luciferase was measured using a dual-luciferase reporter assay system (Promega Corporation) and Mithras LB940 device (Berthold Technologies, Bad Wildbad, Germany) according to the manufacturer’s instructions. Each sample was run at least in triplicate.

### 3.12. Real-Time Monitoring of Cell Proliferation

A549 cells were transfected with miRNA precursors or knockdown oligonucleotides for miR-574-3p (10 or 20 nM) or siRNA for ERH or negative control oligonucleotides (5 nM), as described above. After 24 h of transfection, the cells were trypsinized, counted, and re-plated at a density of 1 × 10^4^ cells/well into a 16-well device compatible with a real-time cell electronic sensing (RT-CES) analyzer and 16X station (Acea Biosciences, San Diego, CA, USA) [72]. Cell growth was monitored for 72 h at 1-h intervals by calculating the cell index (surface area covered by the cells) for each well. The measurements were performed at least in triplicate.

### 3.13. Statistical Analysis

Unless otherwise stated, statistical evaluations of significance were performed using Student’s *t*-tests (two-tailed), with data considered significant when *p* < 0.05.

## 4. Conclusions

In this study, we provide evidence that X-ray-induced upregulation of miR-574-3p resulted in suppression of ERH protein expression and led to a delay in cell growth. Although further studies are necessary to elucidate the precise mechanism of miR-574-3p and its target gene *ERH*, our findings lend important insights into the cellular responses to X-ray irradiation.

## Figures and Tables

**Table 1.** Potential candidate genes regulated by miR-574-3p.

Gene symbol	Gene description	Accession no.	Expression level (signal intensity)			
			FC	KD	OE	*p*-value
ERH	Enhancer of rudimentary homolog (*Drosophila*)	NM_004450.1	−2.1	31,602	14,698	3.8 × 10^−36^
ZYG11A	zyg-11 homolog A (*C. elegans*)	XM_001133615.1	−2.1	269	126	4.5 × 10^−22^
GPR172B	G protein-coupled receptor 172B	NM_001104577.1 (var. 1) NM_017986.3 (var. 2)	−7.1	232	33	0
ZMAT3	Zinc finger, matrin type 3	NM_022470.2 (var. 1) NM_152240.1 (var. 2)	−5.5	80	15	0
ATPAF-AS1	ATPAF1 antisense RNA 1 (non-protein coding)	NM_001145474.1	−3.1	46	13	0.0006
SLC34A1	Solute carrier family 34 (sodium phosphate), member 1	NM_003052.3	−3.8	26	7	4.5 × 10^−9^
AQP7	Aquaporin 7	NM_001170.1	−2.0	24	12	9.7 × 10^−7^
PRDM7	PR domain containing 7	NM_001098173.1 (var. 1) NM_052996.2 (var. 2)	−2.3	20	9	7.0 × 10^−5^

Abbreviations: var. = splicing variant, FC = fold change, KD = knockdown, OE = overexpression. FC was calculated using the “weight by error” method in Resolver software (Rosetta Biosoftware, Seattle, WA, USA).
